# Coupling effects among elementary polarization properties

**DOI:** 10.1038/s41598-020-79174-5

**Published:** 2021-01-14

**Authors:** Wanrong Gao

**Affiliations:** Department of Optical Engineering, Nanjing University of Science and Technology, 200 Xao Ling Wei, Nanjing, 210094 Jiangsu People’s Republic of China

**Keywords:** Biophysics, Optics and photonics

## Abstract

In this work, we propose that there exist coupling effects among birefringence, dichroism and off-diagonal depolarization parameters of differential Mueller matrix of random anisotropic media. An anisotropic spatial correlation function of anisotropic random medium is proposed to explain this phenomenon. The consequences of these effects are then pointed out. The idea in this work is very helpful for accurate interpretation of the measured Mueller matrices of optically anisotropic depolarizing medium. In addition, the concept of the anisotropic spatial correlation function of anisotropic random medium will open a new door and will play a central role for analyzing polarized light scattering by anisotropic random media.

## Introduction

Over the past several decades, the general expressions of both the macroscopic and differential Mueller matrix for nondepolarizing (deterministic) and depolarizing (random) media have been derived from their definitions, simply by using the relationship between the Jones matrix and Mueller matrices, or from the requirements of being physically admissible^1–9^.

The polarization properties of optically anisotropic depolarizing continuous media such as tissues can be described by both the macroscopic and differential Mueller matrices. The macroscopic Mueller matrix can be used when the polarization properties of the total volume is of interest or when only the averaged polarization properties of a volume of tissue sample can be measured. The differential Mueller matrix must be employed when the anisotropic properties of the medium at each position within the medium and their variations with propagation distance are both important such as change of the birefringence with the thickness of skin.

Up to now, several basic facts about the polarization properties of biological tissues have been recognized. First, the real tissues are continuous depolarizing anisotropic media. So in general, the polarization effects of the medium on the light change with the thickness of the traversed medium. Thus only the differential Mueller matrix formalism is appropriate for accurately describing continuous variations of polarization behaviors of tissue. The basic property of the differential Mueller matrix is that the matrix of a thin layer of the given medium with several polarization properties simultaneously is given by the sum of the differential Mueller matrices each of which represents one type of simple differential optical property^1^. This property was also found by Brown in deriving the differential equation for Stokes vector of a light beam propagating through an anisotropic medium^5^. Brown showed that at any point within the deterministic medium, the differential Mueller matrix has the Lorentz group (LG) symmetry and can be expanded as a weighted sum of the LG generators with the weighting parameters representing the elementary polarization properties.

Second, the real tissues are random anisotropic media. Because a general Mueller matrix has sixteen independent parameters and seven of them have been used to represent the elementary polarization properties of the deterministic media, a direct way for finding the form of the differential Mueller matrix of a random anisotropic medium is to introduce another nine parameters to describe their random behaviors^6^. Recognizing the above mentioned LG symmetry, Ossikovski proposed that the nine parameters can be regarded to represent the uncertainties of the seven elementary polarization properties. This interpretation is based on the fact that at the differential level, there exist correspondence between the matrix positions of the elements of the differential Mueller matrix and the elementary polarization properties of anisotropic media, i.e., elements representing the elementary optically anisotropic properties are in the mutually exclusive positions^1^. Note that here three parameters in the depolarizing matrix represent the diagonal anisotropic depolarization effects along the x–y, ± 45°, and circular axes, respectively.

Finally, it has been found that, unlike the elementary polarization properties which can take arbitrary values^1^, there must be relationships between the off-diagonal parameters that represent the uncertainties of the respective elementary polarization properties and the diagonal anisotropic depolarization parameters^7–9^. Based on the result that a physically admissible Mueller matrix can be expressed as a convex sum (all weights are positive) of nondepolarizing matrices^10^, the explicit expressions for these relationships have been derived^7–9^. However, no further explanation is given about the existence of these relationships.

In the last several decades, several decomposition methods have been proposed and tested for calculating diattenuation, birefringence and depolarization parameters from the measured Mueller matrices^2–4^. It has long been believed that the different types of dichroism and birefringence are independent properties of optically anisotropic media. They are then called the elementary polarization parameters. In this work, we propose that this is true for the deterministic anisotropic media or in the deterministic approximation. For random or depolarizing media, there exist some coupling effects among the elementary polarization properties induced by the spatial correlations of the optically anisotropic properties of the medium. Our analysis was stimulated by the results obtained in Refs.^7–9^.

## Methods

### Coupling effects among the elementary polarization properties

In the differential Mueller matrix theory, all polarimetric effects occur simultaneously and there is no dependence on the order of the component matrices. The matrix for the equivalent effect of all the polarization effects can then be obtained by adding their respective matrices. In the most general case, the form of the differential matrix ${\mathbf{m}}$ for an optically anisotropic depolarizing medium can be expressed as a sum of the nondepolarizing part ${\mathbf{m}}_{nondep}$ and depolarizing part ${\mathbf{m}}_{dep}$:

1 $${\mathbf{m}} = {\mathbf{m}}_{nondep} + {\mathbf{m}}_{dep} ,$$

where

2 $${\mathbf{m}}_{nondep} = \left| {\begin{array}{*{20}c} \alpha & \beta & \gamma & \delta \\ \beta & \alpha & \mu & \nu \\ \gamma & { - \mu } & \alpha & \eta \\ \delta & { - \nu } & { - \eta } & \alpha \\ \end{array} } \right|,$$

in which α is the isotropic absorption, β is the linear dichroism along the x–y laboratory axes, γ is the linear dichroism along the ± 45° axes, and δ is the circular dichroism, η is linear birefringence along the x–y laboratory axes, ν is the linear birefringence along the ± 45° axes, and μ is the circular birefringence. Equation (2) is the general expression derived by Azzam for the differential matrix for nondepolarizing medium^1^.

In the first-order approximation, the expression for the depolarizing part ${\mathbf{m}}_{dep}$ of the differential matrix can be expressed as^9^:

3 $${\mathbf{m}}_{dep}^{\left( 1 \right)} = \left[ {\begin{array}{*{20}c} 0 & {\left( {k_{2} + k_{3} } \right)\eta } & {\left( {k_{1} + k_{3} } \right)\nu } & {\left( {k_{1} + k_{2} } \right)\mu } \\ { - \left( {k_{2} + k_{3} } \right)\eta } & { - \left( {k_{2} + k_{3} } \right)} & {\left( {k_{2} - k_{1} } \right)\delta } & {\left( {k_{1} - k_{3} } \right)\gamma } \\ { - \left( {k_{1} + k_{3} } \right)\nu } & {\left( {k_{2} - k_{1} } \right)\delta } & { - \left( {k_{1} + k_{3} } \right)} & {\left( {k_{3} - k_{2} } \right)\beta } \\ { - \left( {k_{1} + k_{2} } \right)\mu } & {\left( {k_{1} - k_{3} } \right)\gamma } & {\left( {k_{3} - k_{2} } \right)\beta } & { - \left( {k_{1} + k_{2} } \right)} \\ \end{array} } \right],$$

4 $${\mathbf{m}}_{dep}^{\left( 2 \right)} = \left[ {\begin{array}{*{20}c} 0 & {k_{4} \left( {1 - 2\eta } \right)} & { - k_{4} \left( {\nu + \delta } \right) - \nu k_{1} } & { - k_{4} \left( {\mu - \gamma } \right) - \mu k_{1} } \\ { - k_{4} \left( {1 - 2\eta } \right)} & { - 2k_{4} \left( {1 - 2\eta } \right)} & {k_{4} \left( {\nu + \delta } \right) - \delta k_{1} } & {k_{4} \left( {\mu - \gamma } \right) + \gamma k_{1} } \\ {k_{4} \left( {\nu + \delta } \right) + \nu k_{1} } & {k_{4} \left( {\nu + \delta } \right) - \delta k_{1} } & { - k_{4} \left( {1 - 2\eta } \right) - k_{1} } & 0 \\ {k_{4} \left( {\mu - \gamma } \right) + \mu k} & {k_{4} \left( {\mu - \gamma } \right) + \gamma k_{1} } & 0 & { - k_{4} \left( {1 - 2\eta } \right) - k_{1} } \\ \end{array} } \right].$$

where $k_{i} \left( {i = 1,2,3,4} \right)$ are the parameters in the depolarizing Mueller matrices corresponding to the elementary matrices^9^:

5a $${\mathbf{H}}_{1} = diag\left[ {0,0, - 1, - 1} \right],\;{\mathbf{H}}_{2} = diag\left[ {0, - 1,0, - 1} \right],\;{\mathbf{H}}_{3} = diag\left[ {0, - 1, - 1,0} \right]$$

5b $${\mathbf{H}}_{4} = \left[ {0,0.5,0,0; - 0.5, - 1,0,0;0,0, - 0.5,0;0,0,0, - 0.5} \right].$$

First it should be pointed out that in order to find the physical significances of the results obtained by Devlaminck et al.^9^, we have adopted the notations and sign conventions used by Azzam for the elementary polarization properties in Eqs. (3) and (4). With the help of forms of expressions (3) and (4) and by using the fact that the elementary optical properties are in the mutually exclusive matrix positions, the coupling effects can be identified and a new concept is proposed to explain them.

Consider the differential Mueller matrix ${\mathbf{m}}_{dep}^{\left( 1 \right)}$ for the type I macroscopic Mueller matrices^11,12^. One can see that the uncertainties of the linear dichroism along the x–y axes $\Delta \beta$, and along the ± 45° axes $\Delta \gamma$ as well as the circular dichroism $\Delta \delta$ are generated exactly due to fluctuations of the linear birefringence along the x–y axes, the linear birefringence along the ± 45°axes, and the circular birefringence, respectively. At the same time, the uncertainties of the linear birefringence along the x–y axes $\Delta \eta$ , the linear birefringence along the ± 45° axes $\Delta \nu$, and the circular birefringence $\Delta \mu$ are induced by the corresponding type of dichroism (see Table 1). Note also that the respective contribution is also linearly related to the parameters $k_{i} \left( {i = 1,2,3} \right)$.

**Table 1:** Coupling effects between elementary polarization properties contained in the differential Mueller matrix for the type I macroscopic Mueller matrices.

Elementary properties	$\Delta \beta$	$\Delta \gamma$	$\Delta \delta$	$\Delta \eta$	$\Delta \nu$	$\Delta \mu$
$\beta$				$(k_{3} - k_{2} )\beta$		
$\gamma$					$(k_{1} - k_{3} )\gamma$	
$\delta$						$(k_{2} - k_{1} )\delta$
$\eta$	$(k_{2} {\text{ + k}}_{3} )\eta$					
$\nu$		$(k_{1} + k_{3} )\nu$				
$\mu$			$(k_{1} + k_{2} )\mu$			

Now we consider the differential Mueller matrix ${\mathbf{m}}_{dep}^{\left( 2 \right)}$ for the type II macroscopic Mueller matrices (Eq. (4)). Equation (4) shows that in this case, the coupling effects are more complex. First, from the first row of the matrix ${\mathbf{m}}_{dep}^{\left( 2 \right)}$ one observes that the fluctuations of the three types of birefringence contribute to the uncertainties of the three types of dichroism. Second, the fluctuation of the circular dichroism also contributes to the uncertainty of the linear dichroism along the ± 45°axes. At the same time, the fluctuation of the linear dichroism along the ± 45°axes also contributes to the uncertainty of the circular dichroism. Third, the uncertainty of the linear birefringence along the x–y laboratory axes is zero. However, it is interesting to note that for the type II macroscopic Mueller matrices, at the differential level, the fluctuation of the linear birefringence along the x–y laboratory axes has a significant contribution to the three diagonal anisotropic depolarization parameters (see Table 2). As a comparison, for the type I macroscopic Mueller matrices, the fluctuations of any of the six elementary polarization properties do not affect the three diagonal anisotropic depolarization parameters.

**Table 2:** Coupling effects between elementary polarization properties contained in the differential Mueller matrix for the type II macroscopic Mueller matrices.

Elementary properties	$\Delta \beta$	$\Delta \gamma$	$\Delta \delta$	$\Delta \eta$	$\Delta \nu$	$\Delta \mu$
$$\beta$$						
$\gamma$			$k_{4} \gamma$		$( - k_{4} + k_{1} )\gamma$	
$\delta$		$- k_{4} \delta$				$(k_{4} - k_{1} )\delta$
$\eta$	$k_{4} (1 - 2\eta )$					
$\nu$		$- (k_{4} + k_{1} )\nu$				$k_{4} \nu$
$\mu$			$- (k_{4} + k_{1} )\mu$			

It is worth noticing that the physical meanings of various types of the coupling effects are uncertainties of the six elementary polarization properties resulting from the depolarization. Here the coupling effects are introduced to emphasize that the uncertainty of each elementary polarization property is “coupled” with the presence of other types of polarization properties. Compared with the elementary polarization properties of nondepolarizing media, these are unique characteristics of the depolarizing media. For example, the uncertainties of the linear dichroism along the x–y axes $\Delta \beta$ is coupled to the fluctuations of the linear birefringence along the x–y axes.

### Anisotropic spatial correlation function of the medium

It is well-known that, in the first order scalar approximation (or the Born approximation), the light scattered along any direction is determined by the spatial correlation function of the isotropic refractive index^13,14^. Furthermore, there is an intimate relationship between the coherence and the polarization of a light beam^15,16^, predicted by Gao in biological tissues^17–19^ and experimentally observed^20^.

In order to explain the coupling effects between the polarization effects, we generalize the spatial correlation function of the isotropic refractive index to the spatial correlation function of the anisotropic refractive indices. Consider a light beam propagating over a distance $d$ into the anisotropic medium. The two orthogonal components of the light electric vector can be expressed as.

6a $$E_{x} \left( d \right) = E_{x0} \exp \left\{ { - j\left[ {\left( {{{2\pi d} \mathord{\left/ {\vphantom {{2\pi d} \lambda }} \right. \kern-\nulldelimiterspace} \lambda }} \right)\left( {n_{x} - j\alpha_{x} } \right)} \right]} \right\},$$

6b $$E_{y} \left( d \right) = E_{y0} \exp \left\{ { - j\left[ {\left( {{{2\pi d} \mathord{\left/ {\vphantom {{2\pi d} \lambda }} \right. \kern-\nulldelimiterspace} \lambda }} \right)\left( {n_{y} - j\alpha_{y} } \right)} \right]} \right\},$$

where $E_{x0}$ and $E_{y0}$ are two components of the light incident on the surface of the sample along the two principal axes ($x$ and $y$) of the medium, $n_{i} \left( {i = x,y} \right)$ are the principal indices of refraction and $\alpha_{i} \left( {i = x,y} \right)$ are the principal extinction coefficients. Note that, in general, the parameters $n_{i} \left( {i = x,y} \right)$ and $\alpha_{i} \left( {i = x,y} \right)$ are functions of the position $\vec{r}$ within the medium. Note that in Eq. (6) we neglect the temporal dependence of the electric vector.

The anisotropic spatial correlation function of the medium can be defined as

7 $${\mathbf{C}} = \left( {\begin{array}{*{20}c} {n_{x} - j\alpha_{x} } \\ {n_{y} - j\alpha_{y} } \\ \end{array} } \right)\left( {\begin{array}{*{20}c} {n_{x} - j\alpha_{x} } & {n_{y} - j\alpha_{y} } \\ \end{array} } \right)^{ * } ,$$

where * stands for complex conjugate. The real tissues are random anisotropic media and its polarization effects fluctuate over the volume. The statistical averages are required. Let $\delta n_{i} \left( {i = x,y} \right)$ denote the varying parts of the refractive indices along the $x -$ and $y -$ axes with $\left\langle {\delta n_{i} } \right\rangle = 0\left( {i = x,y} \right)$, and $\delta \alpha_{i} \left( {i = x,y} \right)$ denote the variations of the principal absorption coefficients along the $x -$ and $y -$ axes with $\left\langle {\delta \alpha_{i} } \right\rangle = 0\left( {i = x,y} \right)$, respectively, we have

8 $$\begin{aligned} {\mathbf{C}} & = \left\langle {\left( {\begin{array}{*{20}c} {\left( {n_{x} + \delta n_{x} } \right) - j\left( {\alpha_{x} + \delta \alpha_{x} } \right)} \\ {\left( {n_{y} + \delta n_{y} } \right) - j\left( {\alpha_{y} + \delta \alpha_{y} } \right)} \\ \end{array} } \right)\left( {\begin{array}{*{20}c} {\left( {n_{x} + \delta n_{x} } \right) - j\left( {\alpha_{x} + \delta \alpha_{x} } \right)} & {\left( {n_{y} + \delta n_{y} } \right) - j\left( {\alpha_{y} + \delta \alpha_{y} } \right)} \\ \end{array} } \right)^{ * } } \right\rangle \\ & = \left( {\begin{array}{*{20}c} {c_{11} } & {c_{12} } \\ {c_{21} } & {c_{22} } \\ \end{array} } \right), \\ \end{aligned}$$

where

9a $$c_{11} = \left\langle {n_{x}^{2} } \right\rangle + \left\langle {\alpha_{x}^{2} } \right\rangle ,$$

9b $$\begin{aligned} c_{12} & = \left[ {\left\langle {n_{x} n_{y} } \right\rangle + \left\langle {\delta n_{x} \delta n_{y} } \right\rangle } \right] + j\left[ {\left\langle {n_{x} \alpha_{y} } \right\rangle + \left\langle {\delta n_{x} \delta \alpha_{y} } \right\rangle } \right] \\ & \quad - j\left[ {\left\langle {n_{y} \alpha_{x} } \right\rangle + \left\langle {\delta \alpha_{x} \delta n_{y} } \right\rangle } \right] + \left[ {\left\langle {\alpha_{x} \alpha_{y} } \right\rangle + \left\langle {\delta \alpha_{x} \delta \alpha_{y} } \right\rangle } \right], \\ \end{aligned}$$

Equations (8) and (9) show that potential spatial correlation between the fluctuations of the anisotropic refractive indices and the absorption coefficients are underlying physical reasons for the existence of several types of coupling effects mentioned above.

The above coupling effects reveal that, at the differential level, statistically there must be some intimate relationships between the three basic types of dichroism and three basic types of birefringence. For the type I macroscopic Mueller matrices, it is shown that the fluctuations of the birefringence will generate the uncertainties of the dichroism along the same axes. The fluctuations of the dichroism will generate the uncertainties of the birefringence along the same axes.

For the type II macroscopic Mueller matrices, in addition to the coupling effects between the elementary dichroism and birefringence, there are also some kinds of coupling effects between different kinds of dichroism or between different kinds of birefringence (see first and second row and column).

### Relate the anisotropic spatial correlation function to the differential matrix

Now we try to relate the anisotropic spatial correlation function ${\mathbf{C}}$ of the medium to the differential matrix ${\mathbf{m}}$. For a thin slab of medium of thickness $\Delta z \to 0$, we have

10 $${\mathbf{M}}\left( {\Delta z} \right){ = }{\mathbf{I}}{ + }{\mathbf{m}}\Delta z,$$

where ${\mathbf{I}}$ is the identity matrix. Kim et al. showed that when an ensemble of transformation is used to represent a random linear medium, an ensemble of Jones matrices corresponds to the Mueller matrix in general^21^. The elements $M_{\mu \nu } \left( {\Delta z} \right)$ of the Mueller matrix ${\mathbf{M}}\left( {\Delta z} \right)$ of the medium of the illuminated volume can be expressed as^21^:

11 $$M_{\mu \nu } \left( {\Delta z} \right) = \frac{1}{2}\left\langle {J_{np}^{\left( e \right)} J_{qm}^{\left( e \right)\dag } } \right\rangle_{e} \sigma_{mn}^{\left( \mu \right)} \sigma_{pq}^{\left( \nu \right)} ,$$

where summation on repeated indices is understood, $\left\langle {} \right\rangle_{e}$ is the shorthand notation for average over the ensemble, † denotes the Hermitian adjoint, $J^{\left( e \right)}$ is a typical element of the ensemble of a 2 × 2 Jones transformation matrix of the thin slab, $\sigma^{\left( \mu \right)}$ ($\mu = 0,1,2,3$) are the four linearly independent 2 × 2 Pauli spin matrices

12 $$\sigma^{\left( 0 \right)} = \left( {\begin{array}{*{20}c} 1 & 0 \\ 0 & 1 \\ \end{array} } \right),\;\sigma^{\left( 1 \right)} = \left( {\begin{array}{*{20}c} 1 & 0 \\ 0 & { - 1} \\ \end{array} } \right),\;\sigma^{\left( 2 \right)} = \left( {\begin{array}{*{20}c} 0 & 1 \\ 1 & 0 \\ \end{array} } \right),\;\sigma^{\left( 3 \right)} = \left( {\begin{array}{*{20}c} 0 & { - i} \\ i & 0 \\ \end{array} } \right)$$

It has also been shown that for anisotropic deterministic medium the electric field vector at $\vec{r} = r\vec{s}$ in the far zone of the scatterer is determined by the Fourier transform of the anisotropic dielectric susceptibility matrix of the medium^22^. Assuming that the incident light beam propagating in a direction specified by a unit vector $\vec{s}_{0}$, we have^22^

13 $$\left( {\begin{array}{*{20}c} {E_{sx}^{\left( \infty \right)} \left( {\vec{r},\omega } \right)} \\ {E_{sy}^{\left( \infty \right)} \left( {\vec{r},\omega } \right)} \\ \end{array} } \right) = \int_{{V\left( {\vec{r}^{\prime}} \right)}} {\left( {\begin{array}{*{20}c} {\eta_{11} \left( {\vec{r}^{\prime},\omega } \right)} & {\eta_{12} \left( {\vec{r}^{\prime},\omega } \right)} \\ {\eta_{21} \left( {\vec{r}^{\prime},\omega } \right)} & {\eta_{22} \left( {\vec{r}^{\prime},\omega } \right)} \\ \end{array} } \right)} \exp \left[ { - j\vec{K} \cdot \left( {\vec{r} - \vec{r}^{\prime}} \right)} \right]d^{3} r^{\prime}\left( {\begin{array}{*{20}c} {E_{x0} } \\ {E_{y0} } \\ \end{array} } \right)a\left( \omega \right)\frac{{e^{{jk\vec{s}_{0} \cdot \vec{r}}} }}{r}\left( {\frac{\omega }{c}} \right)^{2}$$

where $k = {\omega \mathord{\left/ {\vphantom {\omega c}} \right. \kern-\nulldelimiterspace} c}$ is the wavenumber in free space, $a\left( \omega \right)$ is a frequency-dependent variable (for light from a broadband source, $a\left( \omega \right)$ is a random variable), c is the speed of light in vacuo, ω is the angular frequency, $\vec{r}^{\prime}$ denotes the position within the medium, $r$ is the magnitude of the vector $\vec{r} = r\vec{s}$($\vec{s}^{2} = 1$) from the reference point in the medium to the observation point, $\vec{s}$ is a unit vector along the observation direction, $\vec{K} = k\left( {\vec{s} - \vec{s}_{0} } \right)$ , $$\left( {\begin{array}{*{20}c} {E_{x0} } & {E_{y0} } \\ \end{array} } \right)^{T} a\left( \omega \right)e^{{jk\vec{s}_{0} }}$$ is the incident electric field vector, here the superscript T stands for transpose, and $V$ is the illuminated volume. This result is based the first Born approximation to the scattered light and is a generalization of the light scattering properties of the isotropic media^23,24^.

In Eq. (13) the elements of the anisotropic dielectric susceptibility matrix of the medium are defined as^22^

14 $$\eta_{ij} \left( {\vec{r}^{\prime},\omega } \right) = {{\left[ {n_{ij}^{2} \left( {\vec{r}^{\prime},\omega } \right) - 1} \right]} \mathord{\left/ {\vphantom {{\left[ {n_{ij}^{2} \left( {\vec{r}^{\prime},\omega } \right) - 1} \right]} {4\pi }}} \right. \kern-\nulldelimiterspace} {4\pi }}.$$

where $n_{ij}$ is the refractive index along the two principle axes of the medium (for example, $i,j = x,y$).

Equation (13) can formally be rewritten as

15 $$\left( {\begin{array}{*{20}c} {E_{sx}^{\left( \infty \right)} \left( {\vec{r},\omega } \right)} \\ {E_{sy}^{\left( \infty \right)} \left( {\vec{r},\omega } \right)} \\ \end{array} } \right) = \left( {\begin{array}{*{20}c} {J_{11} } & {J_{12} } \\ {J_{21} } & {J_{22} } \\ \end{array} } \right)\left( {\begin{array}{*{20}c} {E_{x0} } \\ {E_{y0} } \\ \end{array} } \right)a\left( \omega \right)e^{{jk\vec{s}_{0} }} .$$

A comparison of Eq. (15) with Eq. (13) shows that the scattered light field vector is proportional to the Fourier transform of the anisotropic dielectric susceptibility matrix of the medium. By using the relation between the refractive index and the anisotropic dielectric susceptibility [see Eq. (14)] it can be shown that the ensemble average of the product of two Jones matrices of an anisotropic random medium in Eq. (11) is then proportional to the Fourier transform of the anisotropic spatial correlation function of the refractive index of the medium^22^. We then have

16 $${\mathbf{M}}\left( {\Delta z} \right){ = }{\mathbf{I}}{ + }{\mathbf{m}}\Delta z = \Xi {\tilde{\mathbf{C}}},$$

where $\Xi$ is a coefficient. From Eq. (16) we have

17 $${\mathbf{m}}\Delta z = \Xi {\tilde{\mathbf{C}}} - {\mathbf{I}}.$$

Equation (17) shows that the differential polarization parameters are directly related to the spatial Fourier transform of the anisotropic spatial correlation function of the medium.

## Some consequences

Several consequences can be expected from these coupling effects. First of all, the presence of the types of coupling effects may arise from specific anisotropic structures which have several types of polarization properties simultaneously and these polarization effects are coupled. So these coupling effects may be used as indicators of the existence of these structures.

Second, it is necessary first to determine the forms of the measured macroscopic Mueller matrix when interpreting it in terms of various types of polarization properties. The correct explanation can then be found by expressing the derived differential Mueller matrices in the form of Eqs. (3) or (4).

Third, for the linear homogeneous media, the Mueller matrix at distance z into the medium is related to differential Mueller matrix in an exponential form^1^. The above coupling effects means that, in general, there are not simple relationships between the elements of a macroscopic Mueller matrix and each polarization parameter, which is a well-known result^2,3^.

In addition, it should be pointed out that, compared with the role of the isotropic spatial function in the scalar light scattering theory^13–21^, the anisotropic spatial correlation function of the medium defined by Eqs. (8) and (9) will open a new door and will play a central role for analyzing scattering of the polarized light by anisotropic random media.

Note that coupling effects induced by linear anisotropic deterministic media between the two transversal orthogonal components of the light vector have long been known and a Jones matrix has been defined by Jones to represent the coupling effects^25–27^. At present, the Jones calculi is the basis for understanding propagation of polarized light through anisotropic deterministic media. The fact that the real biological tissues (for example) are random anisotropic media suggests that the anisotropic spatial correlation function defined in this work can play as least the same role in analyzing polarized light propagation through random anisotropic media.

Note also that the well-known important coupling effects induced by random anisotropic media is the coupling of polarized light to unpolarized light by depolarization, resulting in a decrease of degree of polarization (DOP). DOP is a measure of the polarized portion of a partially polarized beam results from depolarization. The related phenomenon is polarizance which describes the coupling of unpolarized light to polarized light^28^. It has been shown that depolarization of polarized light by biological tissues is correlated to tissue heterogeneity and can reveal clinically relevant features^29,30^.

## Discussions

Notice that with the help of the anisotropic spatial correlation function of the medium it is possible to obtain the spatial correlation or characteristic correlation length inside the anisotropic depolarized complex medium from differential Mueller microscopy and spectroscopy of such medium. Second, the real tissues are random anisotropic media and usually have several types of polarization properties simultaneously. In another words, the diattenuation and birefringence may be caused by the same anisotropic structure in normal biological tissues. For example, the retinal nerve’s fiber layer of the eye presents both birefringence and dichroism^31^. So a model study in the future may help to reveal specific properties of clinical samples.

Note also that in a recent work^32^, three types of coupling effects between the components of the dichroism vector and birefringence vector contained in the measured macroscopic Mueller matrix of deterministic anisotropic medium was considered and the possible consequences of the coupling effects on interpretation of measured Mueller matrices of anisotropic media are clearly pointed out. In the present work, the coupling effects are considered that exist among birefringence, dichroism and off-diagonal depolarization parameters of differential Mueller matrix of random anisotropic media. Furthermore, an anisotropic spatial correlation function of anisotropic random medium is proposed to explain these effects.

Here it should be pointed out that by assuming that each of the values of the elementary optical property be temporally random and fluctuating around its mean value, Devlaminck showed that the uncertainties of the optical properties can be expressed in terms of the correlations between the fluctuations of the complementary optical properties. His results are based on the use of the layered-medium interpretation approach proposed by Jones^27^. He also found that the correlations that exist between the fluctuations of complementary optical properties also contribute to the mean values of the elementary optical properties, which suggests that optically neutral medium at rest may acquire birefringence and dichroism by the occurrence of some forms of fluctuations in the medium^33^.

For a depolarizing medium, its differential matrix ${\mathbf{m}}\left( z \right)$ is random process, the corresponding macroscopic Mueller matrix is then a weighted sum with positive weights of the nondepolarizing Mueller matrices of the medium illuminated by the incident polarized light^34^. By assuming that ${\mathbf{m}}\left( z \right)$ is a Gaussian stochastic operator with finite short correlation distance, Devlaminck was able to express the nondepolarizing part and depolarizing part of the differential matrix in terms of the spatial correlations of the elementary polarization properties. His results are valid when the thickness scale of the sample is much larger than the spatial coherence length of the fluctuations.

In addition, by calculating a spatial or a temporal average of Cloude’s coherency matrix of an infinitesimal slab, Ossikovski and Arteaga showed that fluctuations of the six elementary polarization properties about their mean values result in depolarizing part of the differential matrix which increases quadratically with the thickness of the sample^35^. As pointed out by Devlaminck^34^, these results are obtained when the correlation function is assumed to be locally constant. So their results are valid for sample whose thickness is less than the spatial coherence length or for samples with large thickness when the amplitude of the fluctuations are very low.

As discussed above, our results show that in terms of the anisotropic spatial correlation function of the medium, the differential polarization parameters are related to the Fourier transform of the anisotropic spatial correlation function. However, more future work is needed to exploit the potential consequences of these results.

## Conclusions

In conclusion, the types of the coupling effects are identified among birefringence, dichroism and off-diagonal depolarization parameters of differential Mueller matrix of random anisotropic media. An anisotropic spatial correlation function of anisotropic random medium is then defined to explain this phenomenon. At present a basic method for characterizing continuous anisotropic depolarizing media is to employ the elementary polarization parameters defined by the differential Mueller matrices of anisotropic depolarizing media. So the coupling effects and its explanation proposed in this work are of basic importance for the accurate interpretation of the measured Mueller matrices and relate the calculated values of the polarization parameters to specific microscopic anisotropic structures of tissues.
